# An Improved Large-Scale Stress-Controlled Apparatus for Long-Term Seepage Study of Coarse-Grained Cohesive Soils

**DOI:** 10.3390/s21186280

**Published:** 2021-09-18

**Authors:** Chenghao Chen, Shengshui Chen, Shiang Mei, Shaoyang Han, Xian Zhang, Yi Tang

**Affiliations:** 1 Department of Geotechnical Engineering, Nanjing Hydraulic Research Institute, Nanjing 210029, China; chchen@nhri.cn (C.C.); sschen@nhri.cn (S.C.); jgyang@nhri.cn (X.Z.); 2 College of Water Resources and Environmental Engineering, Zhejiang University of Water Resources and Electric Power, Hangzhou 310018, China; meisa@zjweu.edu.cn; 3 College of Civil Engineering, Hohai University, Nanjing 210098, China; hanshaoyang@hhu.edu.cn

**Keywords:** clay–gravel mixture, non-stop water provision, high hydraulic pressure, long-term seepage, stress relaxation

## Abstract

Clay–gravel mixture has been widely used in high embankment dams and understanding its seepage characteristics is critical to dam safety. From the instrumental perspective, the realization of continuous pressurized water supply becomes a key technical challenge, significantly restricting the working conditions replicated in previous seepage apparatuses. To this end, a novel water provision system, relying on parallel-disposed sensor-based pressure devices, was introduced, so that the application of an existing large-scale stress-controlled apparatus can be expanded to long-term seepage tests regarding coarse-grained cohesive soils. Constant-head permeability tests were conducted on original-graded clay–gravel mixtures to investigate their hydraulic properties, incorporating the influence of stress relaxation. Test results show that with 35% gravel content, the clay–gravel mixture is suitable for dam construction as the core material. The stress relaxation holds a marginal effect on the hydraulic conductivity of soil. The functionality of this improved apparatus is verified, especially under long-term seepage conditions.

## 1. Introduction

Seepage phenomenon, which is regarded as water flowing within continuous void spaces from a point of high energy to a point of low energy, can be found ubiquitously in embankment dams and other water-retaining structures [1,2,3]. The relevant seepage control is of vital importance, given the high proportion of seepage-related dam breach accidents [4]. This issue has become increasingly severe, since the projected dam height in China has rapidly climbed up to 300 m [5].

Among various factors associated with uprising dam height, the hydraulic factor plays an essential role in triggering the seepage erosion [6,7]. Apparatuses that can be used to assess the hydraulic stability have been tremendously fostered, and some take into account the influence of stress states [8,9,10,11]. However, the majority of them lack the capacity to accommodate coarse-grained dam filling material. A few apparatuses tailored for large specimens are technically constrained from realizing the high hydraulic gradient, mainly because of the difficulty of producing highly stable hydraulic pressure. The utilization of pressure pumps and air compressors are alternative methods to address this problem [12,13].

Clay–gravel mixture has been widely utilized as the core wall material in high embankment dams (e.g., 295 m Lianghekou dam and 314 m Shuangjiangkou dam in China). Compared to other rock-fill materials, the core wall material is exposed to a higher hydraulic gradient as a result of relatively lower hydraulic conductivity [5]. Understanding the seepage behavior of this cohesive soil, under high hydraulic gradient in particular, inevitably demands the provision of a long-term stabilized high hydraulic head [14]. With the disadvantages of either the rough control of water pressure or the discontinued water supply, the aforementioned methods are hardly reliable in the case of seepage tests regarding coarse-grained cohesive soil. Meanwhile, stress relaxation is categorized as a time effect on the stress–strain behavior [15]. Considering the long-lasting infiltration process within the dam, stress relaxation may hold an impact on the seepage characteristics of soil by changing the porous voids. However, this issue has not been explicitly studied.

As a consequence, an improvement in sensor-based water provision systems, which allows the accurate water head control of up to 3 MPa and the uninterrupted water circulation, is introduced by component. Its functionality and compatibility with previously designed large-scale stress-controlled apparatuses are evaluated by repeatability tests involving broad-grained cohesive soil. A series of seepage tests on wide-graded clay–gravel mixtures are presented under different stress conditions. The hydraulic influence of stress relaxation is preliminarily discussed based on test results.

## 2. Improved Test Apparatus

### 2.1. Briefing of Existing Apparatus

**Figure 1:**
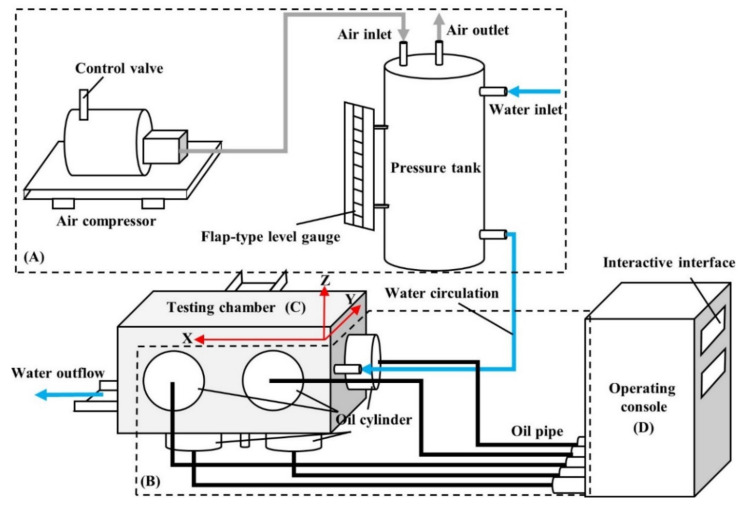
Schematic layout of the existing test apparatus.

Devised by Nanjing Hydraulic Research Institute, the proto-design of the seepage apparatus involved in this study was adopted for implementing high-level hydro-mechanical coupled seepage tests of a variety of soils, especially those containing coarse particles with a constriction size of 75 mm. The schematic illustration of this apparatus is presented in Figure 1. It consists of (A) a water provision system for generation and maintenance of high water pressure, (B) an independent rigid loading system enabling the replication of complex stress states, (C) a large-scale testing chamber that averts the application of the scale-reduction approach on test soil, and (D) an operating console to manipulate stress loading as well as data recording.

The specific instrumental parameters are listed in Table 1. More detailed information along with necessary apparatus settings can be referenced in a previous work [12].

### 2.2. Novel Water Provision System

By using a control valve to regulate the threshold pressure generated by the air compressor, the existing water provision system holds the capability of providing a stable upper water head. However, such a pressurized-air-induced method requires a closed environment within the pressure tank [12]. In other words, it is almost impossible to supply water because of high air pressure, unless the seepage experiment involving high water head is interrupted or terminated. Thus, the volume capacity of the pressure tank greatly restricts the seepage test cycles of soil specimens. This problem becomes increasingly decisive when hydraulic patterns of cohesive soil are under investigation over a longer time period compared to cohesionless soil, which is necessitated to realize a stable flow regime.

**Figure 2:**
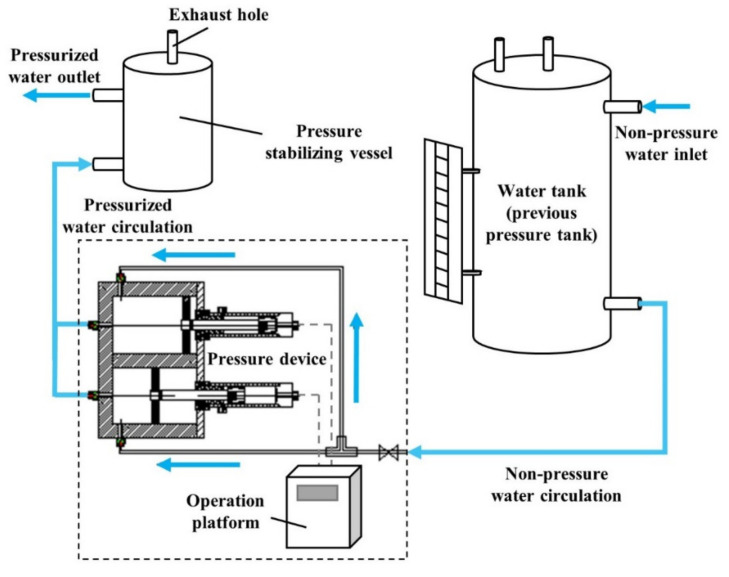
General disposition of the novel water provision system.

**Figure 3:**
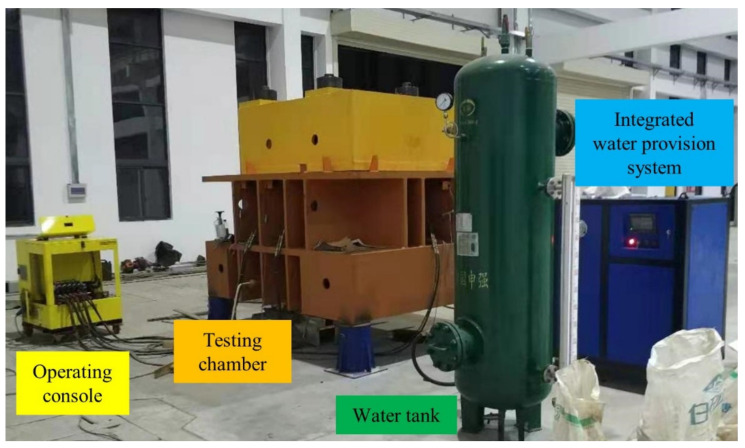
Photograph of the improved large-scale stress-controlled apparatus.

To this end, a renovation of the water provision system was programmed so that the inlet water circulation can stand as a non-stop procedure and continuously sustain the desired water pressure. A different layout, comprised of a water tank, a set of pressure devices, a pressure stabilizing vessel and an operation platform, is displayed in Figure 2. A photograph of this improved apparatus, with the pressure device set and the operation platform integrated as an entity for managing purposes, is shown in Figure 3. The water tank holds a typical reutilization of previously deployed pressure tanks, yet its feature of withstanding high pressure is no longer activated. The external water source flows directly into the tank, providing pressure-free inflow to the distributed pressure device. The magnetic flap level gauge attached to the lateral facet of the tank is not dismantled and contributes continuously to the revealing of water storage during the test.

**Figure 4:**
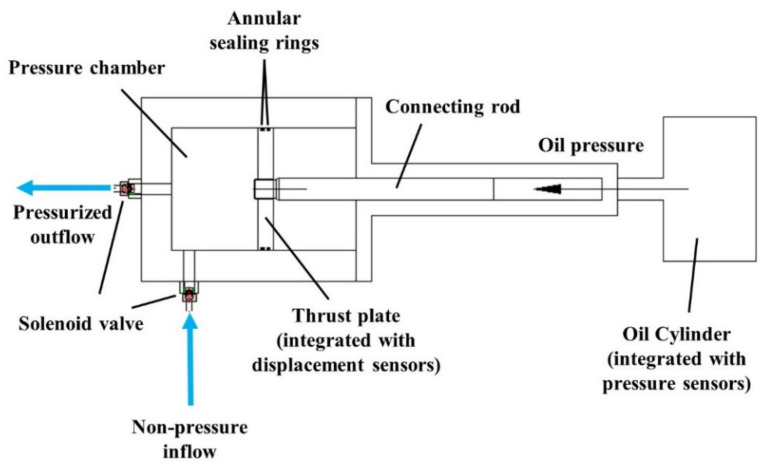
Schematic design of the pressure device.

The independent pressure device, which enables the reproduction of high water pressure, represents the core technical means of water provision improvement. A schematic diagram of the relevant disposition is illustrated in Figure 4. A movable thrust plate powered by an oil cylinder is installed within the pressure chamber of the device, and a pair of high-speed solenoid valves is assembled at both the entrance and the exit of each pressure device. This combined setting is capable of realizing high water head and offering water supply with divergent control instructions. Specifically, under pressure mode, the solenoid valve governing the water inflow is deactivated whilst the outflow valve is activated. In the meantime, the thrust plate is driven to move forward, allowing water in the pressure chamber to be pressurized and transmitted into the pressure stabilizing vessel. When the pressure device is switched to the supply mode, the status of both valves is reversed and the thrust plate is synchronically retracted so that water can be suctioned into the chamber. It is worth noting that two layers of annular sealing rings are embedded at the contact surface between the thrust plate and the sidewall of pressure chamber. This disposition can effectively prevent pressurized water from flowing into the back space of the plate, thus jeopardizing the functionality of the thrust plate, according to preliminary sealing tests.

**Figure 5:**
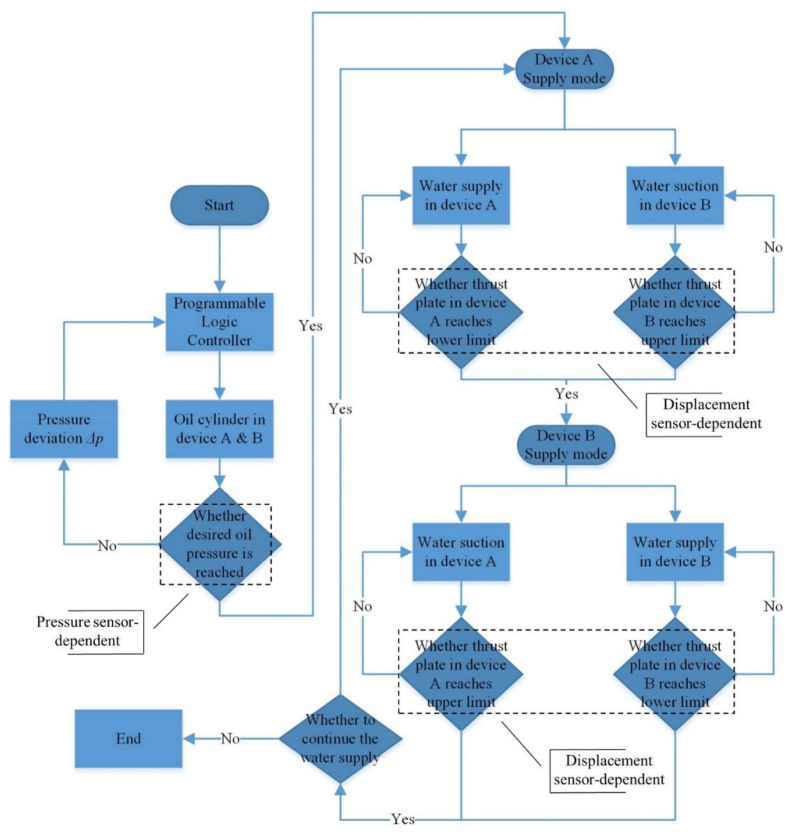
Flow chart of the novel water provision system.

Following the parallel strategy, two pressure devices are disposed with their mode determined by a controlling algorithm. This decision-making algorithm relies on vital information collected from multiple sources, including displacement sensors attached to the thrust plate and pressure sensors monitoring the oil pressure, to control both solenoid valves as well as the oil cylinder. Specifically, the pressure sensor is designed for instantaneous monitoring of oil pressure, which can be converted to water pressure, as well as giving instructions on real-time manipulation of the oil cylinder so that the oil pressure can be maintained at a desired level. The displacement sensor, which is attached to the thrust plate, receives the displacement information of the moving plate. By conveying this information to the Programmable Logic Controller (PLC), the mode of both pressure devices can be promptly switched, prior to potential drop-dead halt and concomitant mechanical loss. A flow chart, which comprises the necessary details regarding the functionality of various sensors as well as the organizational framework of the dual pressure device (referred to as device A and device B), is presented in Figure 5. During the cyclic episodes of seepage tests, there is always one pressure device functioning as the water compressing unit. Meanwhile, the other device conducts the water supply operation until the displacement of the moving plate resets to zero, preparing for the forthcoming shift to the pressure mode. In this way, pressurized water is successively generated and conveyed to the pressure stabilizing vessel.

A container is designed to alleviate the fluctuation of water pressure at the interval when pressure devices are instructed to switch their mode. To evacuate air from the vessel prior to seepage tests, an exhaust hole is fixed on top and kept open until water overflows from the hole. After flowing through the vessel, pressurized water finally circulates into the testing chamber, providing highly stable water pressure to the soil specimen.

**Figure 6:**
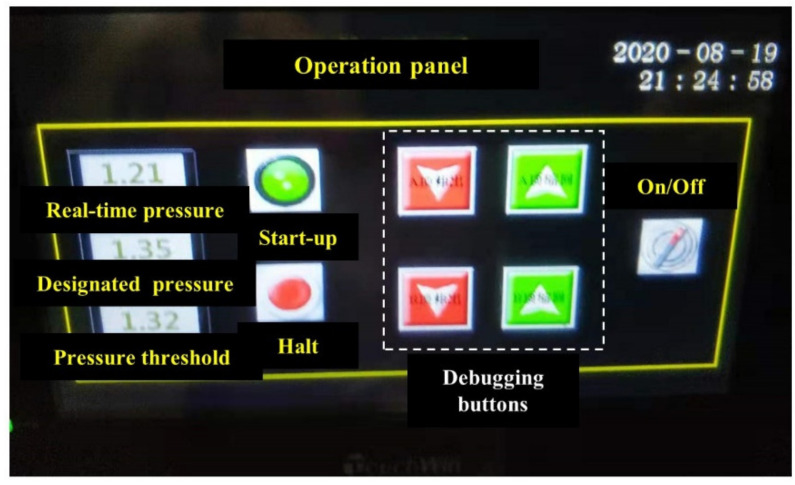
Photographs of the interactive interface.

The operation platform, as demonstrated in Figure 6, is a touchable interactive interface that regulates the power-on/power-off of the entire water provision system. Apart from that, multiple functions are integrated into the operation platform, including the capacity to manipulate the desired water pressure, the sensor-based monitoring of real-time water pressure, and the manual control of both thrust plates under commission and maintenance.

## 3. Materials and Methods

**Figure 7:**
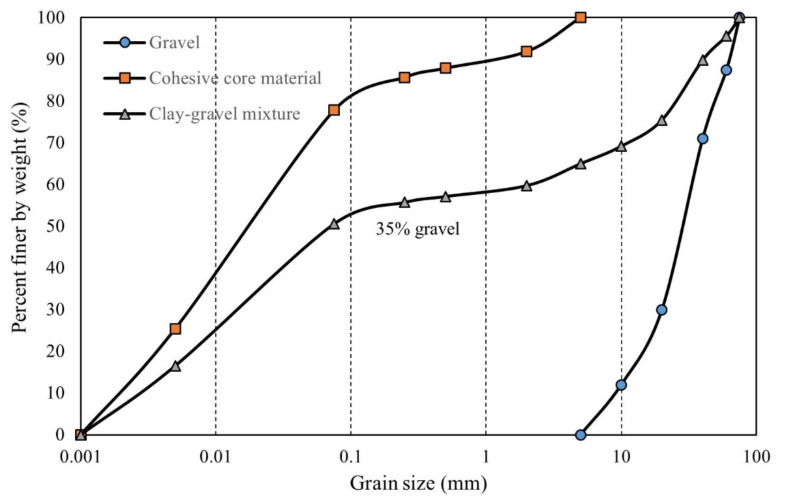
GSD curve of original-graded test material.

The clay–gravel mixtures tested in this study were excavated from 293 m Lianghekou Dam in Sichuan Province, China. The soil matrix is cohesive core material and the coarse part is gravelly soil extracted from Wazhigou stone quarry, 2.5 km away from the dam site. Test cohesive soil, comprised of illite-montmorillonite as its major mineral, is a typical sandy lean clay (contains approximately 78% silty/clayey fines) with a low liquid limit. The level of organic matter, soluble salt, pH as well as dispersity satisfy the requirements of engineering use. Physical properties and the grain size distribution (GSD) curve of core materials are shown in Table 2 and Figure 7, respectively. The gravelly soil is mainly composed of fresh sand-slate under weak weathering and weak unloading conditions. A reconstitution of gravel samples (as shown in Figure 7) was conducted following the mean GSD curve obtained from in situ dry sieving tests, given the fact that gravelly soil had already been sieved. Mixtures with a gravel content of 35% were selected in accordance with the authentic engineering arrangements performed in Lianghekou Dam [16]. As can be seen in Figure 7, the GSD curve of the mixture indicates that no scale-reduction method is required.

In all following tests in this work, the direction of infiltration water remains horizontal. This hydraulic condition is identical to the flow factor in previous experimental studies using this apparatus. Since the renovated water provision system proposed in this study offers the pressurized water through the same pipe, it can be transplanted into the existing apparatus without structural modification. This suggests that no adjustment with respect to apparatus settings is required during the preparation.

In light of the large volume of the test specimen, remolded soil mixtures were prepared following five identical groups. Dry core soil was evenly divided, into which a predetermined amount of water was added to acquire the optimum water content of 17.9%. After being sealed for an equilibration period of 24 h, core soil was thoroughly mixed with the gravel portion (35% by weight of dry soil). Then, the specimen was prepared layer by layer. At the end of each soil layer filling, the mixture was statically compacted to a controlled thickness of 110 mm. The soil surface was chiseled to avoid particle segregation between layers [17]. Subsequently, the back-pressure method was implemented to saturate the specimen. A confining pressure of 0.1 MPa was applied prior to the exertion of upstream water pressure equivalent to 0.05 MPa [18], considering the in situ seepage failure tests. This infiltration period lasted more than 72 h until water continuously overflowed from the top of the specimen. Afterwards, the specimen was enclosed and settled for another 24 h. Preliminary saturation tests show that the soil specimen prepared following the aforementioned saturation procedures can be well saturated.

## 4. Results and Discussion

### 4.1. Repeatability Tests

**Figure 8:**
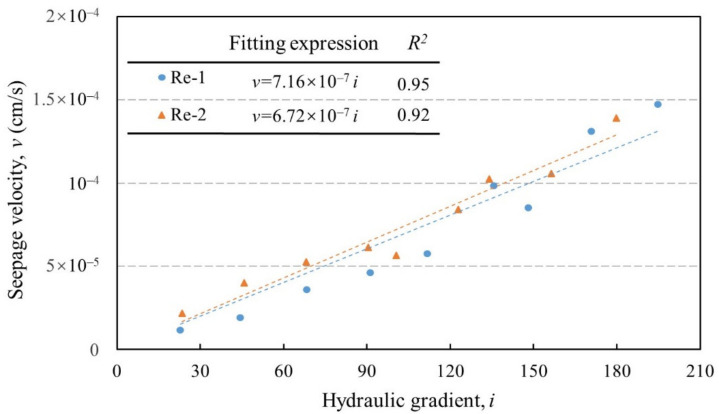
v–i relationship during the repeatability tests.

A total of two independent permeability tests (Re-1 and Re-2) was carried out to evaluate the effectiveness of the newly designed water provision system. Influence factors, including the GSD and the preparation setup, were strictly examined and controlled. Both tests were under the same equi-compressional stress state (*σ_x_ = σ_y_ = σ_z_ =* 0.5 MPa, with the stress orientation defined in Figure 1). The relationships between the flow velocity (*v*) and the hydraulic gradient (*i*) are displayed in Figure 8.

As can be seen, the seepage velocity augments according to the hydraulic gradient increase in Re-1. This changing pattern can be identified as linear-related. A similar relationship between the velocity and the hydraulic gradient is recorded in Re-2, revealing that Darcy’s law remains applicable when the original-graded soil specimen is subjected to seepage flow, irrespective of the addition of gravel particles (35%). The hydraulic conductivity (*k*), which represents the capability of water to infiltrate through a given medium, can be deduced from the slope of the v–i curve. Test results show that the hydraulic conductivity of specimens in the primary and the repeatable tests are 6.72 × 10^−7^ and 7.16 × 10^−7^ cm/s, respectively.

Meanwhile, it is noticeable that the maximal hydraulic gradient loaded to the specimen in both tests reached 194.7 and 179.8, respectively. During this progressive gradient-rising seepage process, neither the abrupt changes of velocity nor the concurrent phenomena describing seepage failure (e.g., turbid outflow, particle dispersion) were observed. These signs infer that no internal erosion, regardless of transient-stage or global-stage, was triggered in repeatability tests [19]. Together with the v–i curve and the hydraulic conductivity determined, a holistic similarity of the hydraulic features of the test soil can be reflected to a great extent. Therefore, the novel water provision system, along with its sensor-relied water pressure control module, is demonstrated to be reliable. The improved apparatus as an entity also shows its suitability for seepage tests lasting a long and uninterrupted time.

### 4.2. Triaxial Seepage Tests Involving Time Effect

Two groups of constant head seepage tests were implemented to investigate the hydraulic properties of soil mixture. The first group involved two comparative tests (CG-1, CG-1sr) under the same normal triaxial stress state, whilst in the second group, the other two tests (CG-2, CG-2sr) were operated under the true triaxial stress state. It is worth remarking that these comparative tests are comprised of a conventional test dominated by coupled hydro-mechanical conditions and a permeability test considering the effect of stress relaxation. The test program is presented in Table 3.

**Figure 9:**
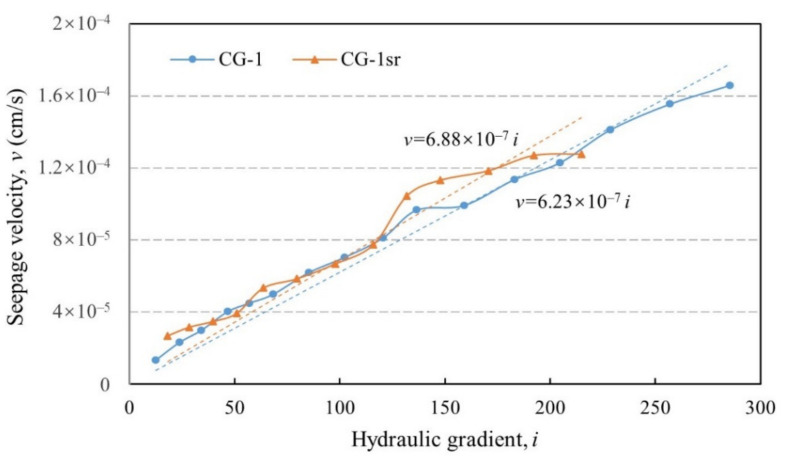
v–i relationships under triaxial stress state (*σ_x_* *= σ_y_ =* 2.5 MPa, *σ_z_ =* 2 MPa).

In test CG-1, the triaxial stress state (*σ_x_* = *σ_y_* = 2.5 MPa, *σ_z_* = 2 MPa) was firstly realized prior to the incremental application of hydraulic pressure. The maintenance of the upstream water head that is produced by the novel water provision system is not interrupted until the measured outflow shows a tendency towards stabilization. Relevant seepage test results are displayed in Figure 9. The flow velocity increases linearly with the hydraulic gradient, representing a typical Darcy flow within the soil mixture. This Darcy flow pattern extends to the condition that the hydraulic gradient exceeds 250. Because of the high coefficient of determination (R^2^ = 0.974) and strong anti-seepage performance, this broad-graded mixture can be regarded as qualified core material in dam construction [20].

The comparative test CG-1sr was carried out under the same initial stress state. However, before the multi-stage water head was imposed onto the specimen, the oil cylinders regulating the stress loading were shut down. In other words, a specimen under stress relaxation condition (fixed volume and gradually decelerated stress) is constituted. A duration of 12 h was controlled, and then the specimen underwent an identical testing procedure. The v–i relationship (see Figure 9) in this scenario agrees with a linear fitting equation with a high precision (R^2^ = 0.927). The corresponding hydraulic conductivity reaches 6.88 × 10^−7^ cm/s, 10.4% larger than that excluding the effect of stress relaxation.

**Figure 10:**
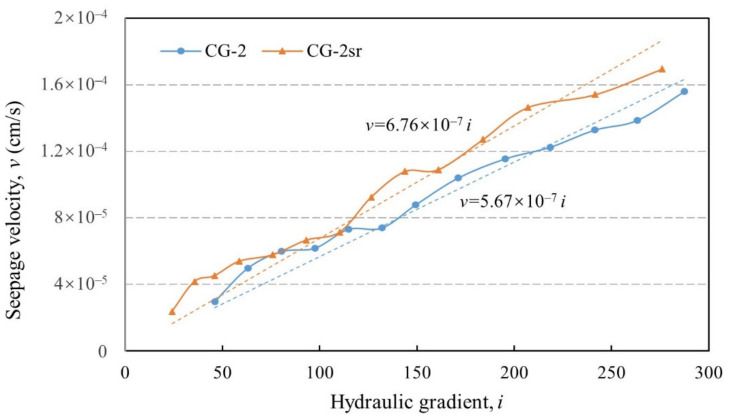
v–i relationships under true triaxial stress state (*σ_x_ =* 3.5 MPa, *σ_y_ =* 2.5 MPa, *σ_z_ =* 4.5 MPa).

The other group of tests were conducted by altering the stress state (*σ_x_ =* 3.5 MPa, *σ_y_ =* 2.5 MPa, *σ_z_ =* 4.5 MPa). The discrepancy of hydraulic behavior induced by stress relaxation is preliminarily probed, given the same amount of relaxation time. As can be seen in Figure 10, the flow velocity increases linearly with the hydraulic gradient (R^2^ = 0.956, 0.948 in test CG-2 and CG-2sr, respectively). Consist with the previous test results, the specimen shows a favorable resistance against seepage failure under a high level of hydraulic gradient, which peaks at 287.5. The hydraulic conductivity in test CG-2 remains the lowest among all permeability tests (including two repeatability tests), achieving 5.67 × 10^−7^ cm/s. This can be attributed to the relatively denser packing state induced by the higher level of confining stress [12,21]. Impacted by a stress relaxation period of 12 h, the permeability of soil mixture in test CG-2sr rises to 6.76 × 10^−7^ cm/s, with an increase equivalent to 19.2%. No indications of seepage failure were witnessed throughout the tests so that the soil matrix consistently maintained its intact form.

From a rheological prospective, stress relaxation can be categorized as one type of time effect on the stress–strain behavior of soils. The restraint on strain deformation, which is accompanied by relaxed stress, can be ascribed to the adjustment of force chain on soil skeleton and the related deformation compatibility of specimens [22]. The structural difference within soil matrix results primarily in the occurrence of stress relaxation. Regarding cohesive soils, the volumetric changes resulting from stress modifications can be divided into plastic deformation and elastic deformation [23]. The deactivation of confining stress leads to the spring-back of the latter form of deformation to a certain extent so that the overall pore size becomes larger. This is in accordance with research carried out with respect to different pore structures influenced by stress relaxation [24]. As a consequence, the hydraulic conductivity displays an upward trend when soils exhibit a relaxation behavior. It is noticeable that the limited magnification of soil permeability as well as the ability to retain soil stability under high hydraulic gradient in this study appear to expound the implication that scarcely a few disturbances of soil skeleton are aroused in the process of stress relaxation. Meanwhile, fine content plays a dominating role on the scour and erosion behavior of the soil [25,26]. Limited skeleton deformation resulting from stress relaxation, as is mentioned above, indicates a relatively subtle variation of constriction size formed among particles. Consequently, the geometric condition, which is an intrinsic parameter to determine the likelihood of internal erosion, appears to undergo an inconspicuous change, and no acute deterioration of anti-seepage performance is witnessed on a global scale.

According to the test results and the aforementioned discussion, the clay–gravel mixture with 35% gravel content shows its applicability as the core-wall material in high dams, as the hydraulic gradient achieved in the tests generally exceed that encountered in real engineering cases. Given the various hydro-mechanical conditions realized by this improved apparatus, a marginal difference (less than 30% deviation) in the hydraulic conductivity of this unconventional soil indicates that a simplified calculation method, which regards the conductivity of clay–gravel mixture as a fixed value, may be applicable for the numerical simulation of seepage. Concerns should be aroused when abrupt changes to downstream dam leakage are observed, as the stress relaxation appears to be a less probable culprit. Further seepage erosion tests combined with stress relaxation control need to be conducted, with a specific focus on the micro-scale observation techniques, e.g., particle image velocimetry (PIV) to monitor porous seepage flow, scanning electron microscope (SEM) to reveal the sample meso-structure subject to stress relaxation, to confirm the aforementioned interpretations. In this way, pore changes induced by stress relaxation can be intuitively displayed, thus enabling the theoretical establishment of a relationship between hydraulic conductivity and stress relaxation.

## 5. Conclusions

A novel water provision system was developed and integrated into an existing large-scale stress-controlled apparatus to study the long-term seepage characteristics of broad-graded cohesive soil under variant hydraulic gradients. This sensor-relied system features continued high-pressure water supply into the testing chamber, which is essential to seepage experiments involving coarse-grained cohesive soils. The extreme pressure that can be smoothly generated reaches 3 MPa, and the related control accuracy is 0.01 MPa. Coordinated with multiple pressure devices, the operating platform transmits different instructions to the oil cylinder and solenoid valves, depending on a collection of pivotal sensing information. The plausible reproduction of uninterrupted high hydraulic pressure, together with the inherent capacity to achieve high stress, displays its feasibility to study the seepage mechanism of embankment dams and infrastructure, particularly on the aspect of long-term seepage behaviors.

Following the horizontal flow orientation, a series of tests under multi-stage hydraulic gradients and different stress states were reported using this improved apparatus. A specific dam material, referred to as the clay–gravel mixture, was utilized. The identification of a Darcy pattern between the seepage velocity and the hydraulic gradient in all tests verifies the suitability of the soil mixture as a core material. The hydraulic conductivity decreases with the increasing stress level, and the stress relaxation holds a peripheral effect on increases in soil permeability. Given the failure to detect internal erosion in comparative tests, the influence of stress relaxation on seepage behavior is not significant and requires further experimental investigation. In a nutshell, these findings can sufficiently prove the reliability of the renovated water provision system and its compatibility with existing large-scale stress-controlled apparatuses.

## Figures and Tables

**Table 1:** Summary of specific instrumental parameters of the existing test apparatus.

Index	Parameter
External size	2000 × 3000 × 2000 mm
Maximum specimen size	1050 × 550 × 550 mm
Minimum specimen size	900 × 450 × 450 mm
Loading form	Rigid loading
Loading direction	3-way directions
Maximum stress level	9 MPa
Maximum water pressure	3.0 MPa

**Table 2:** Index properties of base core material and clay–gravel mixture.

Index	Core Material	Clay–Gravel Mixture (35% Gravel)
Liquid limit, *w_L_*	35.4%	27.0%
Plasticity limit, *w_P_*	15.8%	14.1%
Plasticity index, *I_P_*	19.6	12.9
Specific gravity, *G_s_*	2.75	2.76
Mean particle size, *d*_50_ (mm)	0.019	0.074
Coefficient of uniformity, *C_u_*	-	628.4
Coefficient of curvature, *C_c_*	-	0.145
Maximum dry density, *ρ_d_*_max_ (g/cm^3^)	1.78	2.06
Optimum moisture content, *ω*_op_	17.9%	12.4%

**Table 3:** Summary of seepage tests under various stress states involving time effect.

Test Condition	CG-1	CG-1sr	CG-2	CG-2sr
*σ_x_* (MPa)	2.5		3.5	
*σ_y_* (MPa)	2.5		2.5	
*σ_z_* (MPa)	2		4.5	
*p* (MPa)	2.33		3.5	
*SR* period (h)	-	12	-	12

Note: *σ_x_*, *σ_y_* and *σ_z_* denote the principal stress along *x*, *y* and *z* axis, respectively; *p* denotes the effective mean stress; *SR* period denotes the time interval of stress relaxation prior to seepage test.

## Data Availability

Not applicable.
